# Disposition, metabolism and mass balance of [^14^C]apremilast following oral administration

**DOI:** 10.3109/00498254.2011.604745

**Published:** 2011-08-23

**Authors:** Matthew Hoffmann, Gondi Kumar, Peter Schafer, Dorota Cedzik, Lori Capone, Lai Kei-Fong, Zheming Gu, Dennis Heller, Hao Feng, Sekhar Surapaneni, Oscar Laskin, Anfan Wu

**Affiliations:** 1 Drug Metabolism and Pharmacokinetics Department, Celgene, Summit, NJ, USA; 2 Translational Development Department, Celgene, Summit, NJ, USA; 3 Accellient Partners LLC, Berkeley, CA, USA; 4 XenoBiotic Laboratories, Plainsboro, NJ, USA; 5 R2D Pharma Services LLC, Princeton, NJ, USA; 6 Exploratory Clinical Pharmacology Department, Celgene, Summit, NJ, USA

**Keywords:** Apremilast, human, metabolism, phosphodiesterase type 4, tumour necrosis factor

## Abstract

1. Apremilast is a novel, orally available small molecule that specifically inhibits PDE4and thus modulates multiple pro- and anti-inflammatory mediators, and is currently under clinical development for the treatment of psoriasis and psoriatic arthritis.The pharmacokinetics and disposition of [^14^C]apremilastwas investigated following a single oral dose (20 mg, 100 uCi) to healthy male subjects.
2. Approximately 58% of the radioactive dose was excreted in urine, while faeces contained 39%. Mean C_max_, AUC_0_ and t_max_ values for apremilast in plasma were 333 ng/mL, 1970 ng*h/mL and 1.5 h. Apremilast was extensively metabolized via multiple pathways, with unchanged drug representing 45% of the circulating radioactivity and <7% of the excreted radioactivity.
3. The predominant metabolite was *O*-desmethyl apremilast glucuronide, representing 39% of plasma radioactivity and 34% of excreted radioactivity. The only other radioactive components that represented >4%of the excreted radioactivity were *O*-demethylated apremilast and its hydrolysis product. Additional minor circulating and excreted compounds were formed via *O*-demethylation, *O*-deethylation, *N*-deacetylation, hydroxylation, glucuronidation and/or hydrolysis. The major metabolites were at least 50-fold less pharmacologically active than apremilast. Metabolic clearance of apremilast was the major route of elimination, while non-enzymatic hydrolysis and excretion of unchanged drug were involved to a lesser extent.

## Introduction

**Figure 1:**
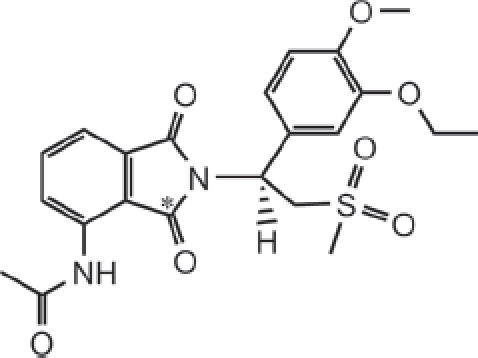
Structure of apremilast, with the site of the ^14^C label indicated (*).

Apremilast [CC-10004; (+)-*N*-[2-[(lS)-1-(3-ethoxy-4-methoxyphenyl)-2-(methylsulfonyl)ethyl]-1,3-dioxo-2,3-dihydro-l*H*-isoindol-4-yl]acetamide] (Figure 1) is an oral agent that inhibits the activity of phosphodiesterase type 4 (PDE4) and the production of multiple pro-inflammatory cytokines and chemokines *in vitro,* including tumour necrosis factor (TNF)-α, interleukin (IL)-8, IL-12, IL-23, CXCL9, CXCL10 and interferon-y (Man et al. 2009; Schafer et al. 2010). Apremilast has demonstrated anti-inflammatory effects *in vitro* and has shown efficacy in a pre-clinical mouse model for psoriasis (Schafer et al. 2010). Additionally, apremilast has shown clinical efficacy in subjects with moderate-to-severe psoriasis (Papp et al. 2008). Apremilast is also under clinical development for the treatment of other inflammatory autoimmune disorders that involve elevated cytokine levels such as psoriatic arthritis and Behcet disease.

The pharmacokinetics of apremilast in patients with severe plaque-type psoriasis following multiple daily doses showed rapid absorption (T_max_=2h) and a moderately long half-life (8.2 h) (Gottelieb et al. 2008). Co-administration of apremilast with ketoconazole resulted in a 36% increase in apremilast AUC (Wu et al. 2006), not only indicating that CYP3A4/5 metabolism plays an important role in apremilast clearance but also suggesting that other clearance pathways are present. This study was performed to evaluate the pharmacokinetics, metabolic disposition and mass balance of a single oral suspension dose (20 mg, 100 μCi) of [^14^C] apremilast to healthy male subjects.

## Materials and methods

### Standards and reagents

[^14^C]Apremilast (Figure 1) was prepared by Girindus America, Inc (Bergisch Gladbach, Germany). The specific activity, radiochemical purity and chemical purity of the material were 5 μCi/mg, >98% and >98%, respectively. Reference standard for apremilast (99.1% chemical purity) was synthesized by Evotec (Oxfordshire, UK). CC-10047 (M5, *O*-desethyl apremilast), CC-10055 (M7,*N*-deacetyl apremilast), CC-15091 (M1/M2, phthalimide ring hydrolysis products of apremilast), CC-16085 (M3, *O*-desmethyl apremilast), CC-16166 (M12, *O*-desmethyl apremilast glucuronide), CC-16401 (M17, acetamide hydroxy metabolite), CC-16557 (M16, acetamide hydroxyl glucuronide) and CC-16793 (M14, *O*-desmethyl, *N*-deacetyl apremilast glucuronide) were synthesized by the Medicinal Chemistry and Process Chemistry groups at Celgene (Summit, NJ). (3S-*cis*)-(+)-Tetrahydro-3,7adiphenylpyrrolo[2, 1-*b*] oxazol-5(6*H*)-one and β-glucuronidase (S8162) were obtained from SigmaAldrich (Milwaukee, WI). All other reagents and chemicals were obtained from commercial sources.

### Study design and dose administration

This was an open-label, inpatient, single-dose study conducted with six non-smoking healthy male volunteers, aged between 19 and 55 years, with body mass index between 19 and 29kg/m^2^. Before initiation of the study, the protocol and consent form were reviewed and approved by the institutional review board. All study participants gave written informed consent before the screening process was initiated. This study was conducted in full accordance with the Declaration of Helsinki and Good Clinical (GCP) as required by and described in 21 Code of Federal Regulations.

Each subject was administered a single oral suspension of 20 mg (100 μCi) of [^14^C] apremilast in distilled water (240mL total volume). Subjects were fasted for 8 h prior to and 4 h following dose administration. The residual radioactivity in the dosing vials was determined and accounted for approximately 0.3% of the total radioactivity. Dose administration, sample collection, sample processing and determination of total radioactivity were conducted at MDS Pharma Services (Lincoln, NE). Determination of apremilast in plasma, metabolite profiling and metabolite characterization were performed at XenoBiotic Laboratories, Inc. (Plainsboro, NJ).

### Sample collection

Urine was collected pre-dose, 0-4, 4-8, 8-12, 12-24 and every 24 h thereafter up to 216h (9 days) following dose administration and was stabilized with one volume of pH 1.5 Sorensen's citrate buffer (25-mM sodium citrate buffer adjusted to pH 1.5). Individual faecal samples were collected pre-dose and for up to 9 days following dose administration, and homogenized using approximately three volumes by weight of pH 1.5 Sorensen's citrate buffer. Blood (two 5-mL samples for plasma radioactivity counting, a 3-mL sample for whole blood radioactivity counting, a 2-mL sample for apremilast analysis and a 10-mL sample for metabolite profiling) was collected into heparinized tubes at pre-dose, 0.5, 1, 1.5, 2, 2.5, 3, 4, 6, 8, 12, 16, 24, 36, 48, 72, 96, 120, 144 and 168 h following dose administration. Blood samples collected for radioactivity counting were stored at or below -20°C until analysed. Plasma was harvested by centrifugation from all other blood samples. The plasma samples for apremilast analysis and metabolite profiling were mixed with an equal volume of pH 1.5 Sorensen's citrate buffer containing 20-μM amastatin. All urine, faecal homogenates and plasma samples were stored at or below -20°C until analysed.

### Radioanalysis

All radioactivity determinations were performed using a Tri-Carb model 1600TR liquid scintillation counter (PerkinElmer, Wellesley, MA). For plasma and urine analysis, duplicate samples of a known volume were mixed with Ultima Gold XR scintillation cocktail and directly analysed by liquid scintillation counting. For faecal homogenate and blood samples, duplicate aliquots were weighed, allowed to dry and combusted using a PerkinElmer model 307 sample oxidizer. The resultant [^14^C]CO_2_ was trapped in Carbosorb (PerkinElmer) in combination with Permafluor and assayed by liquid scintillation counting. For all matrices, an appropriate quench curve was used to convert cpm values to dpm values. Any sample that was less than two times the background dpm was assigned a value of zero. Using these criteria, the lower limit of quantification in plasma, blood, urine and faeces were 2.07 ng [^14^C]apremilast equivalent/mL (ngEq/mL), 2.61 ngEq/g, 1.89 ngEq/mL and 2.52 ngEq/g, respectively.

### Measurement of apremilast in plasma

Plasma concentrations of apremilast were determined using a Chiral liquid chromatography with tandem mass spectrometry (LC/MS/MS) assay validated for concentrations between 1.00 and 1000ng/mL, with quality control (QC) samples prepared at 3.00, 50.0 and 750 ng/mL. Apremilast and (3S-*cis*)-(+)-tetrahydro-3,7a-diphenylpyrrolo[2, 1-*b*] oxazol-5(6*H*)-one (internal standard; IS) were extracted from 50 μL of plasma (stabilized with 50-μL Sorenson's citrate buffer) using liquid-liquid extraction with methyl tertiary butyl ether. The aqueous layer was frozen and the solvent layer was transferred to a new tube. The solvent was evaporated, the samples were reconstituted and injected for LC/MS/MS analysis using a Chiral AGP (150×4.0mm, 5 μm; Chrom Tech, Inc., Apple Valley, MN) analytical column. Positive ions were measured in the multiple reaction monitoring (MRM) mode using a Sciex API-4000 tandem mass spectrometer equipped with a Turbo IonSpray source with precursor→product ion pairs of 461.0→177.9 for apremilast and 280.1→160.3 for IS. For the QC samples, the accuracy ranged from 87.5% to 106.7%.

### Metabolite profiling by high-performance liquid chromatography (HPLC)

Individual plasma samples collected from all subjects at 0.5, 1, 2.5, 8, 24, 36 and 48 h post-dose were analysed for metabolite profiling. A pooled sample (equal volume from 0.5,1,2.5,8 and 24 h) from subject 1 was also used for metabolite profiling. Three volumes of acetonitrile were added to each sample, followed by mixing and centrifu-gation. The supernatant was removed and the pellet was washed twice with acetonitrile. The combined superna-tants were evaporated to dryness under a stream of nitrogen. The residues were re-suspended in acetonitrile: 1% formic acid in water (1:1), assayed for extraction recovery and analysed for metabolite profiling.

For each subject, urine samples were pooled using an equal percentage by volume of the 0-4, 4-8, 8-12 and 12-24-h samples. The 24-48-h urine sample from each subject and a pool of the 0-168-h urine samples from subject 1 were also analysed for metabolite profiling. Samples were mixed and centrifuged prior to the supernatants being transferred to fresh tubes for metabolite profiling analysis.

Faecal homogenates for metabolite profiling were pooled using an equal percentage by weight to generate two pools (0-48 h and 48-96 h) for each subject. An additional pool (0-168 h) was prepared using the samples collected from subject 1. An aliquot (approximately 6 g) of each pool was extracted with three volumes of acetonitrile; samples were mixed and centrifuged. The supernatant was removed and the pellets were extracted twice with an additional 12 mL of acetonitrile each time. The combined supernatants were evaporated to dryness under a stream of nitrogen and the residue was re-suspended in a small volume (approximately 400 μL) of acetonitrile: 1% formic acid in water (1:1). Samples were assayed for extraction recovery prior to metabolite profiling.

HPLC analysis for metabolite profiling was performed using a Shimadzu LC-10ADVP (Shimadzu Corp., Columbia, MD) or a Waters 2695 Alliance Separation Module (Waters Corp., Milford, MA) with the autosampler set to an ambient temperature of approximately 22°C. ASPD-10A VPUV-Vis Detector or a Waters 996 Photodiode array detector set to 254 nm was used to detect UV absorbance. [^14^C]Apremilast and metabolites were separated using a Phenomenex Luna C 18(2) column (150 × 4.6 mm, 3 μm; Phenomenex, Torrance, CA) equipped with a RP-18 guard column (15×3.2 mm, 7 μm) or using an ACE 3 C18 column (150 × 4.6 mm, 3 μm; Advanced Chromatography Technologies, Aberdeen, Scotland) equipped with an ACE 3 guard column (10×3mm, 3 μm). The mobile phases used were 0.4% formic acid in water; pH 3.2 with ammonium hydroxide (mobile phase A) and acetonitrile (mobile phase B). The column temperature was 30°C and the flow rate was 0.7mL/min. The linear gradient was delivered as follows: 0-5 min to 100% A; 5-20 min to 85% A; 20-30 min hold at 85% A; 30-50 min to 65% A; 50-60 min to 60% A; 60-70 min to 50% A; 70-75 min to 0% A; hold at 0% A until 80 min; return to initial conditions over 2 min.

Radioactivity profiles for excreta were determined by radiochromatography using liquid chromatography-accurate radioisotope counting stop-flow system (LC-ARC; AIM Research Company, Wilmington, DE). Radioactivity was detected using a β-Ram Radioflow detector (IN/US Systems, Brandon, FL) equipped with a 1300-μL liquid cell. Liquid scintillation cocktail and column eluate were mixed at a ratio of 2.5:1. Radioactive peaks were quantified using LC-ARC data handling software. Plasma radioactivity profiles were determined by collecting the column eluate into 96-well Deepwell LumaPlates at a rate of 0.25 min/fraction. The plates were dried and the radioactivity in each well determined using a PerkinElmer TopCount NXT Microplate Scintillation Counter. HPLC radiochromatograms were reconstructed using LC-ARC data handling software.

For some urine and faecal samples, extracts were prepared by solid-phase extraction and repeatedly injected, and fractions were collected (0.25min/well) in order to obtain sufficient metabolite concentrations to allow for additional mass spectrometry characterization. The M12 peak isolated from urine was also subjected to β-glucuronidase hydrolysis to confirm its identity. A 2-mL solution of M12 in 0.2-M sodium phosphate buffer (pH 6.7) was incubated with β-glucuronidase (360 units) at 37°C for 4h, and then extracted with ethyl acetate and analysed by LC/MS.

### Metabolite characterization by mass spectrometry

Selected plasma, urine and faecal extracts, as well as isolated metabolites were analysed by HPLC coupled with a radioactivity detector and mass spectrometer. For the mass spectrometry analysis, mobile phase B was changed to methanol, and for some samples, acetonitrile was added as a post-column addition at 0.2mL/min. Minor changes to the LC gradient were also made for some samples to help separate radioactive peaks with similar retention times. Metabolites were characterized using a Finnigan LCQ mass spectrometer (Thermo Scientific, Waltham, MA) or a SCIEX Q Trap (Applied Biosystems, Foster City, CA), equipped with an electrospray source. The Finnigan mass spectrometer was operated in the positive ionization mode, while the SCIEX mass spectrometer was operated in the positive or negative ionization mode. The instrument settings and potentials were adjusted as necessary to provide optimal data. The Finnigan mass spectrometer was operated with an electrospray needle potential of 4.5 kV and a capillary temperature of 240°C. The collision-activated dissociation studies were conducted using helium as the collision gas. For the SCIEX mass spectrometer, the electrospray needle potential was 5.0 kV in the positive mode and 4.5 kV in the negative mode; the turbo probe temperature was 550°C, the curtain gas was nitrogen, the entrance potential was 10 V the declustering potential was 50 V and the collision energy was 8-25 eV.

### PDE4 and TNF-α activity of apremilast metabolites

PDE4 enzyme was isolated from U937 human monocytic cells and used for testing inhibition in a cAMP hydrolysis assay as previously described (Schafer et al. 2010). For the TNF-α production assay, human peripheral blood mono-nuclear cells (PBMC) were isolated from buffy coat of normal donor blood units (Blood Center of New Jersey, East Orange, NJ) by density centrifugation over Ficoll Hypaque (Pharmacia, Piscataway, NJ). Cells were cultured in RPMI 1640 (Life Technologies, Grand Island, NY) supplemented with 5% AB^+^ human serum (Gemini Bio-products, Woodland, CA), 2-mM L-glutamine, 100-U/mL penicillin and 100-μg/mL streptomycin (Life Technologies). PBMC (2×10^5^ cells) were plated in 96-well flat-bottom Costar tissue culture plates (Corning, NY) in duplicate. Cells were stimulated with lipopolysaccharides (LPS) (from *Salmonella abortus equi;* Sigma cat.no. L-1887, St.Louis, MO) at a final concentration of 1 ng/mL in the absence or presence of compounds. The compounds were dissolved in DMSO (Sigma) and subsequent dilutions were done in culture medium immediately before use. The compounds were added to cells 1 h before LPS stimulation. The cells were incubated for 16-18 h at 37°C in 5% CO_2_, and super-natants were then collected, diluted with culture medium and assayed for TNF-α levels by enzyme-linked immunosorbent assay (ThermoFisher, Rockford, IL). Fifty percent inhibitory concentrations (IC_50_) for the PDE4 enzymatic and TNF-α assay were calculated by non-linear regression analysis using Prism 5.1 (GraphPad Software). Regression curves were sigmoidal dose response curves with maximum and minimum constrained to 100% and 0%, respectively.

### Pharmacokinetic analysis

Pharmacokinetic data were generated by non-compartmental analysis of plasma or whole blood versus time profiles using WinNonlin (version 4.1, Enterprise Edition). For individual subjects, the peak concentration (C_max_), time to C_max_ (T_max_), area under the concentration versus time curve from time zero to the last quantifiable concentration (AUC_0-t_), area under the concentration versus time curve from time zero to infinity (AUC_0-∞_ and the apparent terminal elimination half-life (t_½_) were determined. AUC_0-t_) and AUC_0-∞_ were calculated using the linear trapezoidal rule. AUC_0-∞_ was calculated as the sum of AUC_0-∞_ + C_last_/λz, where C_last_, is the last observed quantifiable concentration. The terminal elimination rate constant (λ_z_) and, therefore, t_½_ and AUC_0-∞_ were estimated by fitting a linear regression of log concentration against time values utilizing a minimum of three data points (excluding C_max_) that appeared to be on the terminal elimination portion of the concentration versus time curve. λ_z_, t_½_, and AUC_0-∞_ were not estimated in cases where the terminal elimination phase did not exhibit a linear decline with a regression coefficient (r^2^) > 0.8, or the data points selected did not encompass a time interval of at least one t_½_.

### Calculations

The amount of total radioactivity (ngEq) in urine and faeces was determined by multiplying the volume or weight of the samples by the radioactivity concentration. The dose recovered at each time point was determined by total radioactivity in the sample, divided by the total dose administered, multiplied by 100%. For data values below the limit of quantification, a value of zero was assigned for calculations of means.

## Results

### Excretion of radioactivity

**Figure 2:**
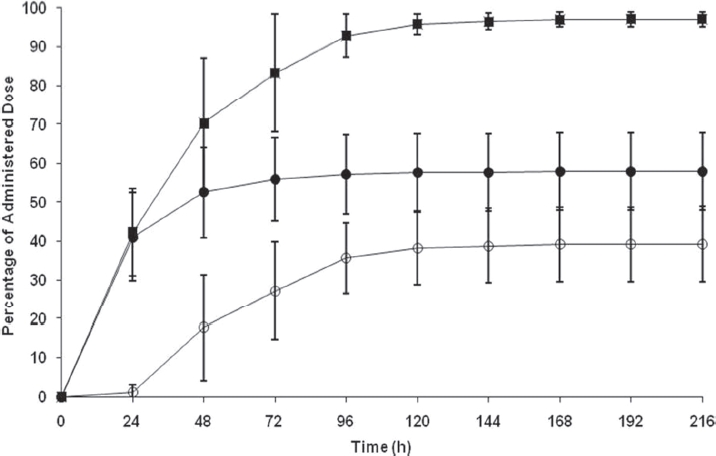
Cumulative elimination of radioactivity in urine and faeces after a single oral 20-mg dose of [^14^C]apremilast in male healthy subjects (ourine, o faeces, ? total). Values are mean ± standard deviation.

Following a single 20 mg, 100-μCi dose of [^14^C]apremilast, urine and faeces were collected from six subjects over 216h (9 days). Excretion of radioactivity was nearly complete, with a mean recovery of approximately 97% (range 94.1%-99.3%). The amount of radioactivity (expressed as percentage of dose) excreted in urine ranged from 47.4% to 71.2% (mean 57.9% ± 9.9%), whereas faecal excretion ranged from 27.6% to 50.8% (mean 39.2% ± 9.7%) over the collection period. The majority of the radioactivity (>90%) was recovered within the first 4 days (96 h) after dose administration (Figure 2).

### Pharmacokinetic analysis

**Figure 3:**
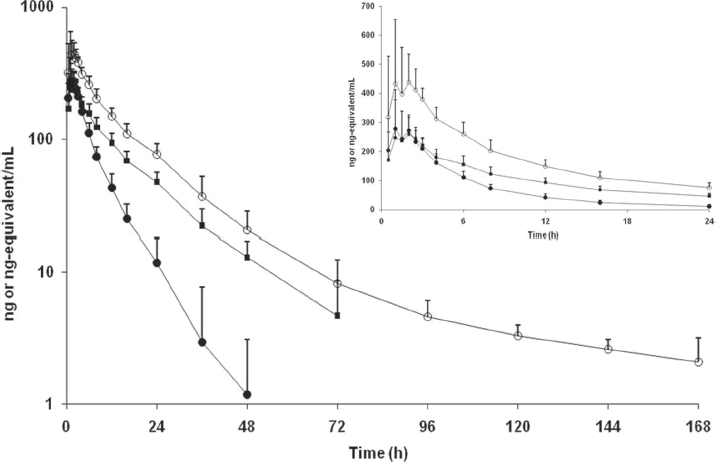
Concentration versus time curves for radioactivity in plasma (○), apremilast in plasma (•) and radioactivity in blood (▪) following a single oral 20-mg dose of [^14^C]apremilast in healthy male subjects. Values are mean ± standard deviation.

**Figure 4:**
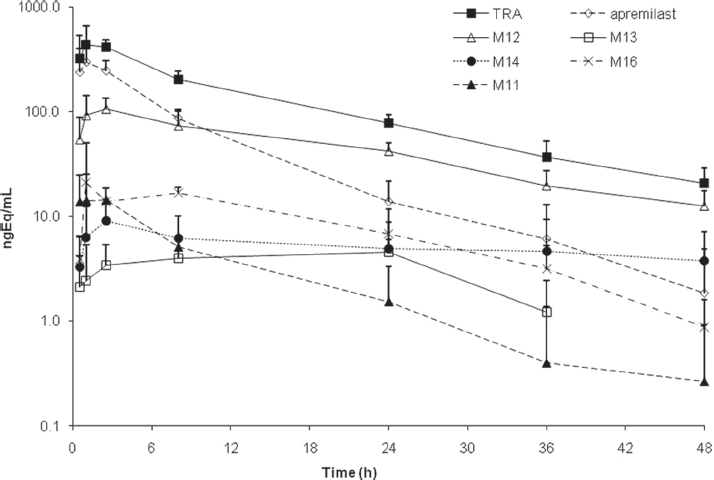
Concentration versus time curves for total radioactivity (TRA), apremilast, Mil, M12, M13, M14 and M16 in plasma following a single oral 20-mg dose of [^14^C]apremilast in healthy male subjects. Values are mean ± standard deviation.

The absorption of [^14^C]apremilast was relatively rapid (T_max_ of 1.5 h) and the plasma half-life was moderate (approximately 7h), while plasma radioactivity had a much longer half-life (approximately 50 h) (Tables 1 and 2, Figure 3), suggesting the presence of metabolites that are longer-lived than parent compound. Apremilast was generally not detected in plasma beyond the 48-h time point, whereas radioactivity was detected out to 168 h following dose administration. Modest differences in apremilast AUC determined via LC/MS (1913±339 ng*h/mL) or via radiochromatography (2455 ±690 ngEq*h/mL) may be attributed to method and limit of detection differences. Based on radiochromatography, plasma exposure (AUC) of apremilast was approximately 40% relative to total radioactivity. The pharmacokinetic parameters of some of the more abundant metabolites were determined using radiochromatography (Table 2, Figure 4). The peak plasma concentrations of the metabolites generally occurred later (T_max_ of 1-5 h for metabolites vs. 1.5 h for apremilast) and the metabolites had somewhat longer t½ values than apremilast (11-16 h for metabolites vs 7 h for apremilast). The most abundant circulating metabolite was M12 (*O*-desmethyl apremilast glucuronide), which represented 39% of the circulating radioactivity and had an AUC similar to apremilast. In addition, nine other metabolites were detectable in plasma, none representing >7% of the circulating radioactivity. The mean blood/plasma ratios of radioactivity were between 0.53 and 0.63 at all time points evaluated (data not shown), indicating no noteworthy binding of radioactivity (apremilast and its metabolites) to the blood cell constituents.

**Table 1:** Plasma and whole blood total radioactivity pharmacokinetic parameters following a single oral 20-mg dose of [^14^C]apremilast.

PK parameter	Whole-blood total radioactivity^a^	Plasma total radioactivity^a^	Blood-to-plasma ratio
C_max_ (ngEq/mL)	303±77	527±127	0.57
T_max_ (h)	2.0(1.0-3.0)	1.5(1.0-3.0)	NA
AUC_0-t_ (ngEq*h/mL)	3489±509	6201±937	0.56
AUC_0-∞_ (ngEq*h/mL)	3664±556	6632±653	0.55
t_½_	16.3±5.2	50.4±8.7	NA

NA, not applicable; ngEq, ng [^14^C]apremilast equivalent.

a Values are reported as mean ± standard deviation except T_max_ values, which are reported as median (min-max).

**Table 2:** Mean ± standard deviation plasma pharmacokinetic parameters for apremilast and circulating metabolites after a single oral 20-mg dose of [^14^C]apremilast.

	TRA	Apremilast^a^	Apremilast^b^	M11	M12	M13	M14	M16
C_max_ (ngEq/mL)	527 ±127	333±76	321±134	20.2±7.6	111±36	7.5±6.8	9.4±4.3	27.6±26.0
T_max_(h)	1.5(1.0-3.0)	1.5(1.0-3.0)	1.8(1.0-2.5)	1.0(0.5-2.5)	2.5(1.0-2.5)	2.5(1.0-24)	2.5(1.0-24)	5.3(1.0-8.0)
AUC_0-t_ (ngEq*h/mL)	5483 ± 825^c^	1913±339	2455±690	139±89	2124±331	133±125	269±146	363 ±54
AUC_0-∞_ (ngEq*h/mL)	6632 ± 653	1970±343	2636±705	232±151	2446±416	n/c^d^	n/c	389±91
t_½_(h)	50.4 ±8.7	6.8±2.6	7.1±2.7	10.7 ±10.2	15.8±3.9	n/c	n/c	11.0±2.4

T_max_, median and range; TRA, total radioactivity.

a Apremilast concentrations in plasma determined using a Chiral LC/MS/MS assay.

b Apremilast and metabolite concentrations in plasma calculated using plasma radioactivity concentrations and radiochromatography.

c AUC_0-48_ was used for TRA for these calculations.

d Not calculated.

### Metabolic profiles

**Figure 5:**
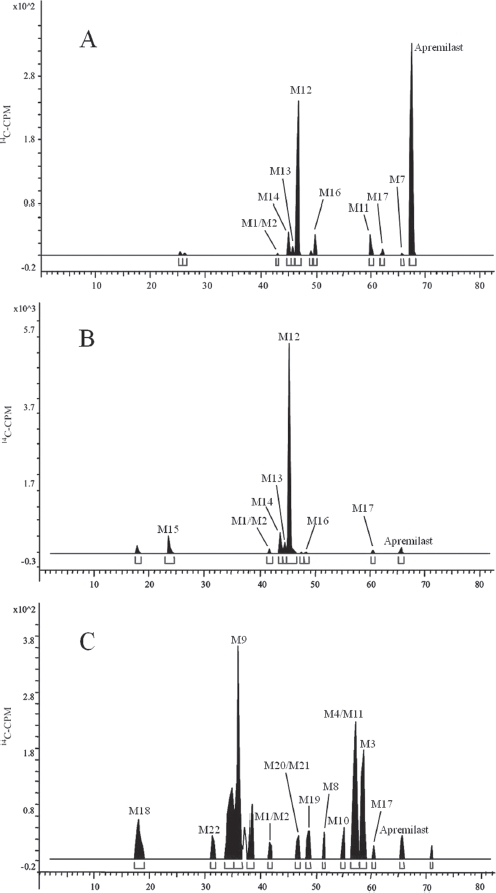
Representative radiochromatograms of (A) 0-24-h pooled plasma, (B) 0-24-h pooled urine and (C) 0-48-h pooled faeces after a single oral 20-mg dose of [^14^C]apremilast in healthy male subjects.

HPLC radiochromatograms of plasma, urine and faecal homogenate from a representative subject are shown in Figure 5. Metabolite profiles were similar between subjects and qualitatively similar at different time points. Apremilast was the predominant radioactive component in plasma up to 8-h post-dose, after which M12 was the predominant compound. By 48-h post-dose, apremilast was not detected in plasma samples of most subjects. In plasma, apremilast plus metabolites M7, M11, M12, M13, M14 and M16 accounted for an average of 94.8% of the radioactivity in the samples.

Apremilast was a minor component in urine collected up to 48-h post-dose, representing <3% of the dose (Table 3). The major urinary metabolite was M12, which accounted for 34% of the dose. An additional nine minor metabolites were observed in urine, each representing ≤4% of the dose. Apremilast was also a minor component in faeces collected up to 96-h post-dose, representing 4% of the dose. The two most abundant faecal metabolites were the *O*-desmethyl metabolite (M3, 5% of the dose) and its hydrolysis product (M9, 8% of the dose). The remaining 14 faecal metabolites each represented ≤3% of the dose. Approximately, 92.4% and 86.0% of the radioactivity in urine and faeces, respectively, were characterized and identified as metabolites listed in Table 3.

**Table 3:** Fragment ions and relative amounts for apremilast metabolites characterized in plasma, urine and faeces.

Metabolite Characterization				Plasma (% AUC relative to TRA)^d^	% of dose excreted	
		
Number	Pathway	[M + H]^+^^a^	Fragment Ions		Urine	Faeces
Apremilast	Unchanged	461	257,205, 178, 163, 150	44.8	2.8	4.1
M1/M2	Hydrolysis products of apremilast	479	274, 257, 223, 206, 178	D	0.9	0.5
M3	*O*-Demethylation	447	243, 164, 136	D	D	4.6
M4	*O*-Demethylation, *N*-deacetylation	405	243,164	ND	ND	2.4
M7	*N*-Deacetylation	419	257, 178, 163	D	D	0.1
M8	Hydroxylation, *O*-demethylation, *N*-deacetylation	421	243, 179, 164	ND	ND	1.0
M9	Hydrolysis product of M3	465	260, 243, 223, 206, 164	ND	ND	7.7
M10	Hydroxylation, *O*-demethylation	463	243,221, 164, 163, 136	ND	ND	1.3
M11	Hydroxylation, *N*-deacetylation	435	257, 179, 178	2.5	ND	1.4
M12	*O*-Demethylation, glucuronidation	623	447, 243	38.7	33.7	ND
M13	*O*-Deethylation, glucuronidation	609	488, 433, 229, 205, 150	2.4	2.0	ND
M14	*O*-Demethylation, *N*-deacetylation, glucuronidation	581	405, 243	4.9	4.0	ND
M15	Hydrolysis product of M12	641	465, 436, 260, 243, 164	ND	3.3	ND
M16	Hydroxylation, glucuronidation	653	477,397,257	6.6	0.6	ND
M17	Hydroxylation	477	257,221, 178, 163	D	1.2	0.9
M18	Hydrolysis	222°	178, 136, 134, 92	ND	ND	1.4
M19	*O*-Demethylation, *O*-deethylation	419	215,205, 163, 136	ND	ND	0.8
M20^b^	*O*-Demethylation, *O*-deethylation, *N*-deacetylation	377	215, 163, 136	ND	ND	0.5
M21^b^	Hydroxylation, *O*-demethylation, *O*-deethylation	435	221,215, 163, 136	ND	ND	
M22	Hydrolysis, *O*-deethylation	451	246, 229, 223, 206, 150, 135	ND	ND	0.6
M23	Hydrolysis, hydroxylation	238^c^	194, 150, 136, 92, 74	ND	ND	3.0

ND, not detected; D, detected by MS, but at concentrations too low to accurately quantify by radiochromatography.

a Many compounds were observed as [M + H]^+^ and [M + NHJ^+^.

b M20 and M21 coeluted under the HPLC conditions used.

c Characterized in negative mode, so values are [M - H]∼

d AUC_0-t_) (unchanged drug or metabolite)/AUC_0___48_ (TRA) * 100%.

### Metabolite characterization

**Figure 6:**
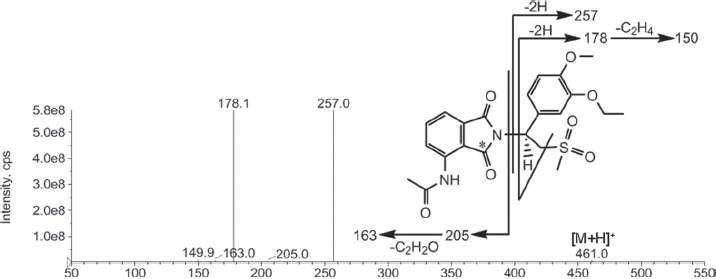
Mass spectral fragmentation of apremilast.

**Figure 7:**
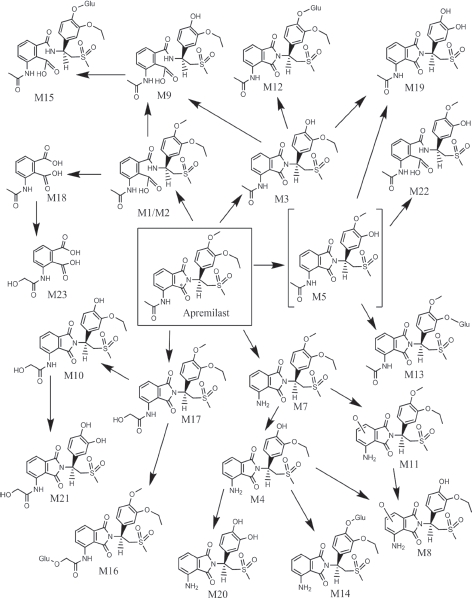
Metabolic scheme of apremilast in humans. For hydrolysed phthalidomide ring products, only one of two possible forms is shown. [M5, *O*-desethyl apremilast, was not observed in this study and is a proposed intermediate metabolite] (GLU: glucuronic acid, * site of ^14^C label).

Figure 6 shows the proposed mass spectrometry fragmentation pattern for apremilast, Table 3 lists the characteristic fragment ions for apremilast and the metabolites, and Figure 7 shows the proposed metabolite structures. Using reference standard, apremilast generated a protonated molecular ion, [M + H]^+^, at *m/z* 461. The product ions of *m/z* 461 mass spectrum included *m/z* 257, 205, 178, 163 and 150. Loss of *N*-dioxoisoindlinyl acetamide generated *m/z* 257. Subsequent loss of hydro-sulphonylmethane generated *m/z* 178 and additional loss of ethane generated *m/z* 150. The *N*-dioxoisoindlinyl acetamide portion of the molecule generated the *m/z* 205, and subsequent loss of C_2_H_2_O from the *N*-acetyl moiety generated *m/z* 163.

Metabolites M1/M2 generated a [M + H]^+^ at *m/z* 479, which was 18 Da larger than apremilast, consistent with hydrolysis products of apremilast. The product ion for M1/M2 at *m/z* 223 was 18 Da greater than apremilast, indicating the *N*-dioxoisoindrinyl acetamide portion of the molecule was the site of hydrolysis. Subsequent loss of NH_3_ yielded *m/z* 206, consistent with hydrolysis occurring on the imide ring. Therefore, M1/M2 were identified as hydrolysis products of apremilast with hydrolysis occurring at either side of the nitrogen on the imide ring. The presence of M1/M2 in plasma was confirmed by LC/ MS/MS in the MRM mode.

The [M + H]^+^ for M3 was observed at *m/z* 447, which was 14 Da lower than apremilast, suggesting loss of a methyl group. Product ions at *m/z* 243, 164 and 136 were also 14 Da lower than those observed for apremilast, consistent with the *O*-demethylation of the methoxy group of the phenyl ring. Using M3 from mouse faeces, the MS/MS spectra for M3 was shown to be consistent with CC-15604 reference standard and, therefore, M3 was identified as *O*-desmethyl apremilast. The presence of M3 in plasma and urine was confirmed by LC/MS/MS in the MRM mode.

The [M + H]^+^ for M4 was observed at *m/z* 405, which was 56 Da lower than apremilast. Product ions at *m/z* 243 and 164 were 14 Da lower than the corresponding ions observed for apremilast, suggesting *O*-demethylation of the methoxy group of the phenyl ring. The [M + H]^+^ for M4 at *m/z* 405 is 42 Da lower than M3, consistent with *N*-deacetylation of *O*-desmethyl apremilast. Metabolite M4 was identified as *N*-deacetylated, *O*-desmethyl apremilast.

The [M + H]^+^ for M7 was observed at *m/z* 419, which is 42 Da lower than apremilast, consistent with *N*-deacetylation. The product ions at *m/z* 257, 178 and 163, as well as the retention time of 63.3 min were consistent with those observed for *N*-deacetylated apremilast reference standard. Using M7 from mouse faeces, the MS/MS spectra for M7 was shown to be consistent with CC-10055 reference standard and, therefore, M7 was identified as *N*-deacetylated apremilast.

Metabolite M8 had a [M + H]^+^ at *m/z* 421, which was 40 Da lower than apremilast. Product ions *m/z* 243 and 164 for M8 were 14 Da lower than the corresponding product ions for apremilast, suggesting *O*-demethylation of the methoxy group on the phenyl ring. The product ion *m/z* 179 for M8 was 26 Da lower than the corresponding product ion for apremilast, suggesting hydroxylation and *N*-deacetylation on the *N*-dioxoisoindlinyl acetamide portion of the molecule. Metabolite M8 was identified as hydroxylated *N*-deacetylated, *O*-desmethyl apremilast.

The [M + H]^+^ for M9 was observed at *m/z* 465, which is 4 Da larger than apremilast. The product ions at *m/z* 243 and 164 were 14 Da lower than the corresponding ions for apremilast, consistent with *O*-demethylation of the methoxy group on the phenyl ring. The product ion for M9 at *m/z* 223 was 18 Da greater than apremilast, suggesting hydrolysis on the *N*-dioxoisoindlinyl acetamide portion of the molecule. Subsequent loss of NH_3_ yielded *m/z* 206, consistent with hydrolysis occurring on the imide ring. Similar product ions were observed for metabolites M1/M2 (hydrolysis products of apremilast). Metabolite M9 was characterized as the hydrolysis product of *O*-desmethyl apremilast.

Metabolite M10 had a [M + H]^+^ at *m/z* 463, which was 2 Da greater than apremilast. Product ions *m/z* 243, 164 and 136 for M10 were 14 Da lower than the corresponding product ions for apremilast, suggesting *O*-demethylation of the methoxy group on the phenyl ring. The product ion *m/z* 221 for M10 was 16 Da greater than the corresponding product ion for apremilast, suggesting hydroxylation on the *N*-dioxoisoindlinyl acetamide portion of the molecule. The product ion for M10 at *m/z* 163 was also observed for apremilast, suggesting the isoindoline-dione portion of the molecule was not the site of hydroxylation. Therefore, M10 was identified as acetamide hydroxylated, *O*-desmethyl apremilast.

The [M + NH_4_]^+^ for M11 was observed at *m/z* 452, corresponding to a molecular weight of 434, which is 26 Da less than apremilast. The product ions at *m/z* 257 and 178 were also observed for apremilast, indicating the *N*-dioxoisoindlinyl acetamide portion of the molecule was the site of metabolism. The product ion for M11 at *m/z* 179, suggested hydroxylation and *N*-deacetylation on the *N*-dioxoisoindlinyl acetamide portion of the molecule. Metabolite M11 was characterized as hydroxylated *N*-deacetylated apremilast. The presence of M11 in plasma was confirmed by LC/MS/MS in the MRM mode.

The [M + NH_4_^+^ for M12 was observed at *m/z* 640, corresponding to a molecular weight of 622, which is 162 Da greater than apremilast. Loss of 176 Da led to the product ion at *m/z* 447 for M12, indicating M12 was a glucuronic acid conjugate. The product ions at *m/z* 447 and 243 for M12 were consistent with *O*-demethylation of apremilast. Additionally, isolation of the M12 radioactive peak followed hydrolysis by β-glucuronidase resulted in a single major radioactive product, which was identified by LC/MS to be metabolite M3. Therefore, M12 was identified as the glucuronide conjugate of *O*-desmethyl apremilast. The presence of M12 in plasma was confirmed by LC/MS/MS in the MRM mode.

The [M + H]^+^ for M13 was observed at *m/z* 609, which is 148 Da greater than apremilast. Loss of 176 Da led to the product ion at *m/z* 433 for M13, indicating M13 was a glucuronic acid conjugate. The product ion at *m/z* 229 was 28 Da lower than the corresponding product ion for apremilast and was consistent with *O*-deethylation of the ethoxy group of the phenyl ring. M13 was identified as the glucuronide conjugate of *O*-desethyl apremilast. The presence of M13 in plasma was confirmed by LC/MS/MS in the MRM mode.

The [M + NH_4_^+^ for M14 was observed at *m/z* 598, corresponding to a molecular weight of 580, which is 120 Da greater than apremilast. The product ion at *m/z* 405 for M14 was consistent with the loss of 176Da from the metabolite, indicating M14 was a glucuronic acid conjugate. The product ions at *m/z* 405 and 243 for M14 were consistent with *N*-deacetylation and *O*-demethylation of apremilast. Therefore, M14 was characterized as the glucuronide conjugate of *N*-deacetylated, *O*-desmethyl apremilast. The presence of M14 in plasma was confirmed by LC/MS/MS in the MRM mode.

The [M + H]^+^ for M15 was observed at *m/z* 641, which is 180 Da larger than apremilast. The product ion at *m/z* 465 indicated the loss of 176 Da, suggesting M15 was a glucuronic acid conjugate. Further fragmentation of the product ion at *m/z* 465 yielded product ions similar to those observed for metabolite M9 (the hydrolysis product of *O*-desmethyl apremilast). Metabolite M15 was, therefore, characterized as the hydrolysis product of the glucuronide conjugate of *O*-desmethyl apremilast.

The molecular weight of M16 was determined to be 652 based on the [M + NH_4_^+^ at *m/z* 670. This molecular weight was 192 Da larger than apremilast, indicative of hydroxylation plus glucuronidation. The product ion at *m/z* 397 was consistent with hydroxylation and glucuronidation on the *N*-dioxoisoindrinyl acetamide portion of the molecule. Additionally, the product ion at *m/z* 257 was also observed for apremilast indicating the *N*-dioxoisoindlinyl acetamide portion of the molecule was the likely site of metabolism. Therefore, M16 was characterized as the glucuronide conjugate of hydroxylated apremilast, with the site of metabolism on the *N*-phenylacetamide portion of the *N*-dioxoisoindlinyl acetamide ring. Using M16 from mouse plasma and bile, the MS/MS spectra for M16 was shown to be consistent with CC-16557 reference standard and, therefore, M16 was identified as acetamide hydroxylation apremilast glucuronide. The presence of M16 in plasma was confirmed by LC/MS/MS in the MRM mode.

The [M + H]^+^ for M17 was observed at *m/z* 477, which is 16 Da larger than apremilast suggesting a hydroxylated metabolite. The product ion at *m/z* 221 was 16 Da greater than the corresponding product ion for apremilast, suggesting the *N*-dioxoisoindlinyl acetamide portion of the molecule was the likely site of metabolism. The product ion at *m/z* 163 was also observed for apremilast indicating that the acetamide was the site of hydroxylation. Using M17 from mouse plasma and bile, the MS/MS spectra for M17 was shown to be consistent with CC-16401 reference standard and, therefore, M17 was identified as hydroxy apremilast, with the acetamide being the site of metabolism. The presence of M17 in plasma was confirmed by LC/MS/MS in the MRM mode.

The [M + H]-for M18 was observed at *m/z* 222, suggesting cleavage of apremilast, with a significant portion of the molecule lost. Based on the molecular weight and the known metabolic pathways for apremilast, the structure of M18 was suggested to be 3-acetamide-phthalic acid. Product ions at *m/z* 178, 136, 134 and 92 were consistent with this structure. Therefore, M18 was characterized as 3-acetamide-phthalic acid.

The [M + H]^+^ for M19 was observed at *m/z* 419, corresponding to a molecular weight of 418, which is 42 Da less than apremilast. The product ions at *m/z* 205 and 163 were also observed for apremilast, indicating the *N*-dioxoisoindlinyl acetamide portion of the molecule was not the site of metabolism. The product ions for M19 at *m/z* 215 and 136 were 42 Da less than the corresponding product ions of apremilast, suggesting *O*-deethylation and *O*-demethylation of the ethoxy and methoxy groups of the phenyl ring. Metabolite M19 was characterized as *O*-desmethyl, *O*-desethyl apremilast.

The [M + H]^+^ for M20 was observed at *m/z* 377, which is 84 Da less than apremilast. The product ions at *m/z* 215 and 136 were 42 Da less than the corresponding product ions of apremilast, suggesting *O*-deethylation and *O*-demethylation of the ethoxy and methoxy groups of the phenyl ring. The product ion for M20 at *m/z* 163 was consistent with *N*-deacetylation. Therefore, M20 was characterized as *N*-deacetylated, *O*-desmethyl, *O*-desethyl apremilast.

The [M + NH_4_^+^ for M21 was observed at *m/z* 452, corresponding to a molecular weight of 434, which is 26 Da greater than apremilast. The product ions at *m/z* 215 and 136, were 42 Da less than the corresponding product ions of apremilast, suggesting *O*-deethylation and *O*-demethylation of the ethoxy and methoxy groups of the phenyl ring. The product ion at *m/z* 221 was 16 Da greater than the corresponding product ion for apremilast, suggesting the *N*-dioxoisoindlinyl acetamide portion of the molecule was the likely site of hydroxylation. The product ion at *m/z* 163 was also observed for apremilast indicating that the acetamide was the site of hydroxylation. Metabolite M21 was, therefore, characterized as hydroxy *O*-desmethyl *O*-desethyl apremilast, with the acetamide being the site of hydroxylation.

The [M + H]^+^ for M22 was observed at *m/z* 451, which is 10 Da less than apremilast. The product ion at *m/z* 229 was 28 Da lower than the corresponding ions for apremilast, consistent with *O*-deethylation of the ethoxy group on the phenyl ring. The product ion for M22 at *m/z* 223 was 18 Da greater than apremilast, suggesting hydrolysis on the *N*-dioxoisoindrinyl acetamide portion of the molecule. Subsequent loss of NH_3_ yielded *m/z* 206, consistent with hydrolysis occurring on the imide ring. Similar product ions were observed for metabolites M1/M2 (hydrolysis products of apremilast). Metabolite M22 was characterized as the hydrolysis product of *O*-desethyl apremilast.

The [M + H]^-^ for M23 was observed at *m/z* 238, which was 16 Da larger than metabolite M18. Based on the molecular weight and the known metabolic pathways for apremilast, the structure of M18 was suggested to be hydroxy 3-acetamide-phthalic acid. Product ions at *m/z* 194, 150, 136 and 92 were consistent with this structure, with hydroxylation occurring on the acetamide portion of the molecule. Therefore, M23 was characterized as hydroxy 3-acetamide-phthalic acid.

### PDE4 and TNF-α activity of apremilast metabolites

The major apremilast metabolites were at least 50-fold less active than apremilast with regard to their ability to inhibit PDE4 and TNF-α (Table 4). The minor metabolites M7 and M17 did retain some PDE4 and TNF-α inhibition activity, with IC_50_ values similar to those of apremilast.

**Table 4:** Phosphodiesterase type 4 and tumour necrosis factor-α inhibitory activities of apremilast and its metabolites.

Compound	PDE4IC_50_(μM)	TNF-α IC_50_(μM)
Apremilast (S-isomer)	0.074	0.077
M1/M2; Hydrolysis products of apremilast (S-isomer)	120	77
M3; *O*-Desmethyl apremilast (S-isomer)	8.3	5.6
M5; *O*-desethyl apremilast (race mate)	44	4.9
M7; *N*-deacetyl metabolite (S-isomer)	0.16	0.13
M12; *O*-Desmethyl apremilast glucuronide (S-isomer)	>100	>10
M14; *O*-Desmethyl, *N*-deacetyl apremilast glucuronide	>80	>10
M16; Acetamide hydroxylation glucuronide (S-isomer)	6.5	>10
M17; Acetamide hydroxylation (S-isomer)	0.094	0.021

## Discussion

Pharmacokinetic analysis of total radioactivity and apremilast following a single oral [^14^C]apremilast dose to healthy volunteers suggests rapid absorption, with plasma T_max_ values <2h. Additionally, the amount of unchanged apremilast in faeces (4% of the dose), suggests absorption of the majority of the apremilast dose in humans. Elimination of radioactivity from blood, as well as apremilast and its major metabolites from plasma, was moderate with half-lives ranging between 7 and 16 h with some metabolites having slightly longer half-lives than apremilast. The one exception being metabolite M14, which had concentrations that were similar in all of the plasma samples analysed between 8 and 48 h and for which a half-life was not calculated. The terminal elimination half-life of total radioactivity from plasma was significantly longer (approximately 50 h) mostly because the lower detection limit in plasma allowed for radioactivity to be detected out to 168 h, as compared with 48 and 72 h for apremilast in plasma and radioactivity in blood, respectively. The long half-life of radioactivity in plasma would likely result in little accumulation because it is based on the concentrations observed in the 96-168-h samples, which were generally 100-fold less than the C_max_ values. As a result, approximately 90% of the AUC_0-∞_ for radioactivity in plasma was between 0 and 96 h, with the remaining (AUC_96-∞_ J accounting for only 10%. This is consistent with the excretion data that showed recovery of radioactivity was fairly rapidly (>92% radioactivity recovered at 96 h) and nearly complete by 216 h (>97%).

The pharmacokinetic parameters reported for apremilast in this study are comparable to previously published data in patients with severe plaque-like psoriasis (Gottlieb et al. 2008). In that study, patients received 20 mg of apremilast daily for 29 days, with pharmacokinetic samples collected following the final dose. Even though samples were collected following multiple daily doses, the T_max_ (2h), C_max_ (207ng/mL) and AUC_0-24_ (1799 ng*h/mL) values reported were similar to those reported in this study suggesting little accumulation of the drug. The difference in C_max_ values between the studies is likely due to the different dosage forms used in the studies, oral suspension in this study and capsules in the multiple dose study.

Apremilast was extensively metabolized in humans, with approximately 7% of the dose excreted unchanged and 45% of the circulating radioactivity identified as apremilast (based on radiochromatography) through 48 h. The predominant circulating and excreted metabolite was M12 (*O*-desmethyl apremilast glucuronide), which accounted for 39% of the circulating radioactivity and 34% of the excreted dose. The other circulating metabolites were formed via a number of pathways, including *O*-demethylation, *O*-deethylation, *N*-deacetylation, hydrolysis, hydroxylation and/or glucuronidation. Since the *O*-demethylation of apremilast is primarily catalysed by CYP3A4 (data not shown), the effects of ketoconazole (a known CYP3A4 inhibitor) co-administration on the pharmacokinetics of apremilast have been evaluated in healthy subjects (Wu et al. 2006). Co-administration of ketoconazole resulted in small but consistent increases in the mean apremilast C_max_ (5% increase) and AUC (36% increase).

Based on urinary radioactivity, at least 58% of the drug was absorbed, and if faecal metabolites are included in the calculation, the amount absorbed may be >80%. The urinary metabolites of apremilast were similar to those observed in plasma, while the metabolites in faeces were primarily the aglycone and/or hydrolysis products of the metabolites observed in circulation, suggesting they were initially formed in the liver, excreted into bile and subsequently cleaved or hydrolysed in the intestine. Hydrolysis of apremilast, and presumably its metabolites, occurs non-enzymatically in solution at neutral pH and possibly enzymatically in plasma and hepatocytes (data not shown). Together with the ketoconazole interaction data, these data suggest that multiple pathways, including CYP3A4-mediated metabolism, non-enzymatic hydrolysis, non-CYP3A4-mediated metabolism and elimination of unchanged drug, play important roles in the clearance of apremilast.

Most of the circulating apremilast metabolites were tested for their PDE4 and TNF-α inhibitory activities and the major metabolites were substantially (>50-fold) less active than apremilast. The minor metabolites M7 and M17 did retain some pharmacological activity but were present in circulation at concentrations that were generally <2% relative to apremilast and, therefore, are unlikely to contribute to the pharmacological activity of apremilast. These data are consistent with work that has been performed evaluating the structure-activity relationship of PDE4 inhibitors (Man et al. 2009; Van der Mey et al. 2001; Kodimuthali et al. 2008). Based on these data, substitutions larger than a methoxy or ethoxy group at the 4-position of the phenyl ring lead to a significant reduction in PDE4 inhibition. Likewise, hydrolysis of the phthalimide ring, as in M1/M2 has been shown to decrease TNF-α inhibition (McHugh & Rowland, 1997), and presumably PDE4 inhibition as well.

In summary, this study demonstrated that apremilast was rapidly absorbed following oral dosing and the majority of the radioactivity was eliminated within 96 h. While the predominant clearance pathway for apremilast was CYP-mediated metabolism, additional clearance pathways also contributed. Although apremilast was one of the major components in plasma, absorbed drug was also extensively metabolized via multiple pathways (*O*-demethylation, *O*-deethylation, *N*-deacetylation, hydroxylation, glucuronidation and/or hydrolysis), with the predominant circulating and excreted metabolite formed via *O*-demethylation followed by glucuronidation. Based on the PDE4 and TNF-α inhibitory effects of the metabolites, it is unlikely that metabolites contribute appreciably to the pharmacological activity of apremilast.

## Declaration of interest

All studies reported here were supported by Celgene and conducted by or under the supervision of Celgene employees. The authors report no conflicts of interest.
